# Anatomical Study of the Extreme Lateral Transpsoas Lumbar Interbody Fusion with Application to Minimizing Injury to the Kidney

**DOI:** 10.7759/cureus.2123

**Published:** 2018-01-29

**Authors:** Joe Iwanaga, Emre Yilmaz, Tamir Tawfik, Amir Abdul-Jabbar, Marc Vetter, Marc Moisi, Koichi Watanabe, Koh-ichi Yamaki, R. Shane Tubbs, Rod J Oskouian

**Affiliations:** 1 Seattle Science Foundation; 2 Swedish Medical Center, Swedish Neuroscience Institute; 3 Neurosurgery, Wayne State University School of Medicine.; 4 Department of Anatomy, Kurume University School of Medicine; 5 Neurosurgery, Seattle Science Foundation; 6 Neurosurgery, Swedish Neuroscience Institute

**Keywords:** anatomy, spine surgery, vertebral column, surgery, complications

## Abstract

Objective

Since the extreme lateral lumbar interbody fusion procedure was first reported by Ozgur in 2006, a large number of clinical studies have been published. Anatomical studies which explore methods to avoid visceral structures, such as the kidney, with this approach have not been examined in detail. We dissected the retroperitoneal space to analyze how the extreme lateral transpsoas approach to the lumbar spine could damage the kidney and related structures.

Methods

Eight sides from four fresh Caucasian cadavers were used for this study. The latissimus dorsi muscle and the thoracolumbar fascia were dissected to open the retroperitoneum. The fat tissue was removed. Steel wires were then put into the intervertebral disc spaces. Finally, the closest distance between kidney and wires on each interdiscal space was measured.

Results

The closest distance from the wire in the interdiscal space on L1/2, L2/3 and L3/4 to the kidney ranged from 13.2 mm to 32.9 mm, 20.0 mm to 27.7 mm, and 20.5 mm to 46.6 mm, respectively. The distance from the kidney to the interdiscal space at L4/5 was too great to be considered applicable to this study.

Conclusions

The results of this study might help surgeons better recognize the proximity of the kidney and avoid injury to it during the extreme lateral transpsoas approach to the lumbar spine.

## Introduction

The minimally invasive retroperitoneal approach to the lumbar spine was first described by Mayer in 1997 [1,2] followed by McAfee, et al. in 1998 [2] and Pimenta (Pimenta L: Lateral endoscopic transpsoas retroperitoneal approach for lumbar spine surgery. Paper presented at: VIII Brazilian Spine Society Meeting. 2001) and Ozgur, et al. [3] in 2006, who first reported the extreme lateral lumbar interbody fusion procedure. This approach is a minimally invasive technique for lumbar fusion and approaches the lateral lumbar spine via the space between the 12th rib and highest point of the iliac crest to enter the retroperitoneal space and through the psoas major muscle to reach the lumbar spine. This approach allows direct access to the intervertebral disc space with no disruption of the peritoneal structures or posterior paraspinal musculature [4-8].

According to Kwon and Kim [9], disadvantages of the lateral transpsoas approach to the lumbar spine include the learning curve associated with new surgical procedures and the orientation of regional retroperitoneal anatomy, which is often unfamiliar to spine surgeons. Complications caused by this approach include neurologic deficits, injuries to abdominal organs and the ureters, or blood vessels [10]. Interestingly, anatomical studies aimed at the position of the kidney in relation to this approach have not been performed.

Although the left kidney is slightly superior to its counterpart, the kidneys are generally located lateral to the psoas major muscle with a superior border located around the level of the 12th thoracic lumbar vertebra and an inferior border near the level of the third to fourth lumbar vertebrae in the retroperitoneal space, making them vulnerable to injury during the lateral transpsoas approach. This is especially true if there are anatomical variants or pathology involving the kidneys or if the operator is unfamiliar with the three-dimensional anatomy of the retroperitoneum. Therefore, we aimed to dissect the retroperitoneal space to analyze how the extreme lateral transpsoas approach to the lumbar spine might damage the kidneys.

## Materials and methods

Eight sides from four fresh Caucasian cadavers (two males and two females with a mean age of 79.5 ± 6.9 years at death) were used for this study. The specimens were placed in the full lateral position and taped to the dissection table. A skin incision was made into the space between the 12th rib and the iliac crest. The underlying musculature and aponeuroses were dissected. The retroperitoneum was exposed. Metal wires were then placed into the intervertebral disc spaces. The placement was confirmed using anteroposterior and lateral fluoroscopy. All wires were placed by fellowship-trained spine surgeons. The wires were positioned at L1/L2, L2/L3, L3/L4 and L4/L5 levels. The closest distance from the wires to the kidney was measured by two different surgeons. The position of the kidney in relation to the lumbar vertebrae was documented. The measurement was carried out twice by two observers for a total of four measurements and then averaged. When the distance was more than 50 mm, it was classified as “not applicable (N/A)” because the risk of kidney injury at such a distance is very low. The protocol of the present study did not require approval by the ethics committees of our institutions and the work was performed in accordance with the requirements of the Declaration of Helsinki (64th WMA General Assembly, Fortaleza, Brazil, October 2013).

## Results

**Figure 1:**
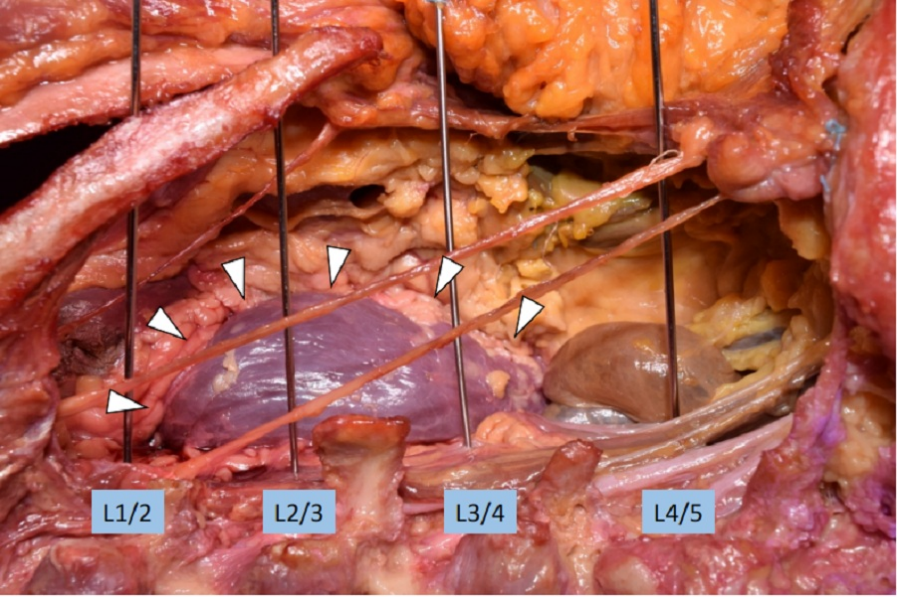
Measurement of the closest distance from the wire for the disc space of L1/2, L2/3 and L3/4 to the kidney (arrowheads).

**Figure 2:**
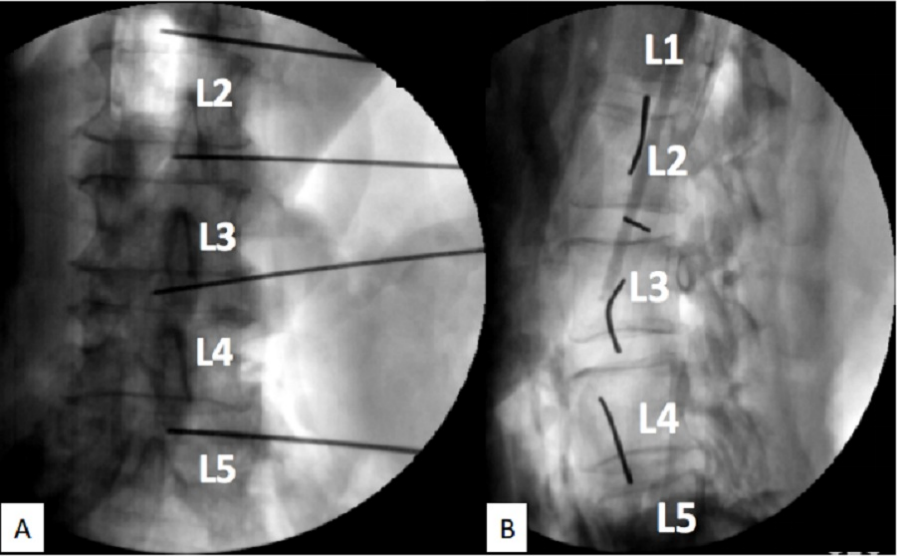
Fluoroscopy of the wire trajectory. Note that all the wires are within disc spaces. A: Lateral view B: Posterior-anterior view

The kidneys were easily identified lateral to the lumbar vertebrae. The closest distance from the wires for the disc space of L1/2, L2/3 and L3/4 to the kidney ranged from 13.2 mm to 32.9 mm (mean 21.1 mm), from 20.0 mm to 27.7 mm (mean 24.5 mm), and from 20.5 mm to 46.6 mm (mean 34.7 mm), respectively (Figures 1, 2). The distance from the kidney to the disc space at L4/5 was not applicable because on all eight sides the distance was greater than 50 mm. No anatomical variants of the kidneys or renal vasculature were identified. No pathological findings such as renal cysts were identified. No specimen had significant abdominal pathological or surgical history in the abdominal area.

## Discussion

The surgical techniques and clinical outcomes of the extreme lateral transpsoas approach to the lumbar spine have been well documented [5, 6, 8, 9]. However, anatomical studies regarding this approach are scant [11-15]. Most of these have focused on neurologic injury [11-15]. Only one report by Voin, et al. [15] has described the anatomical relationships with this procedure and the ureters. To our knowledge, only three cases of iatrogenic renal injury during the extreme lateral transpsoas approach to the lumbar spine have been reported. Blizzard, et al. [16] reported a renal artery injury during the T12-L1 fixation which was successfully identified and treated intraoperatively. Although the details were not included, Isaacs, et al. [17] reported an injury to the kidney with a lateral transpsoas approach. Yuan, et al. [18] reported an injury to the renal vein as a complication of the extreme lateral approach to the lumbar spine. In the present study, the shortest distance to the kidney ranged from 13.2 to 46.6 mm and for left and right sides, the kidney was nearest the operative field at the L1/2 level.

As the position of the kidneys is variable, preoperative imaging to localize their position might decrease the risks of iatrogenic injury during lateral approaches to the lumbar spine. Normally, the right kidney lies between the first and third lumbar vertebrae and the left kidney is slightly lower than the right. Each kidney is approximately 11 cm in length, 6 cm in width and 3 cm in its anteroposterior dimension. The left kidney is often slightly longer than the right kidney [19].

**Figure 3:**
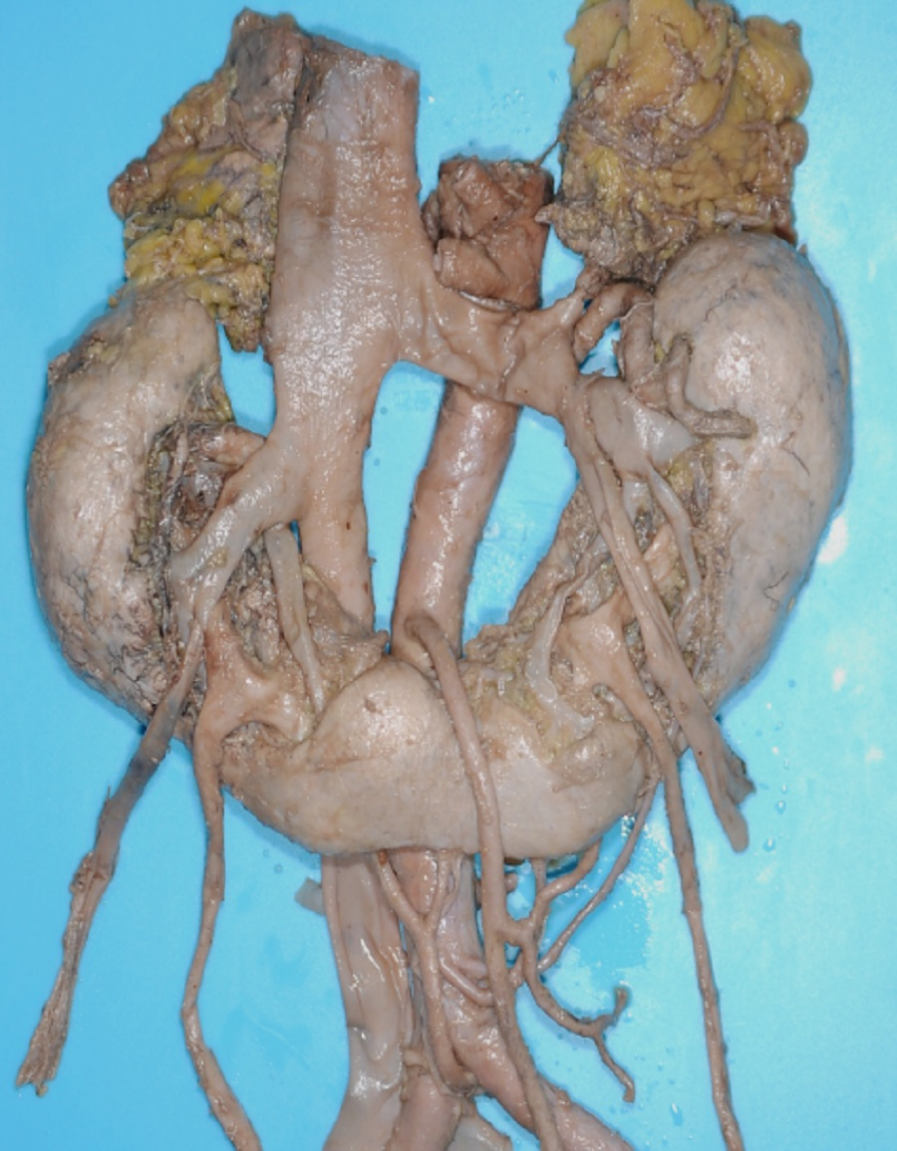
Horseshoe kidney. Slightly lower than normal kidney and often having aberrant renal arteries.

**Figure 4:**
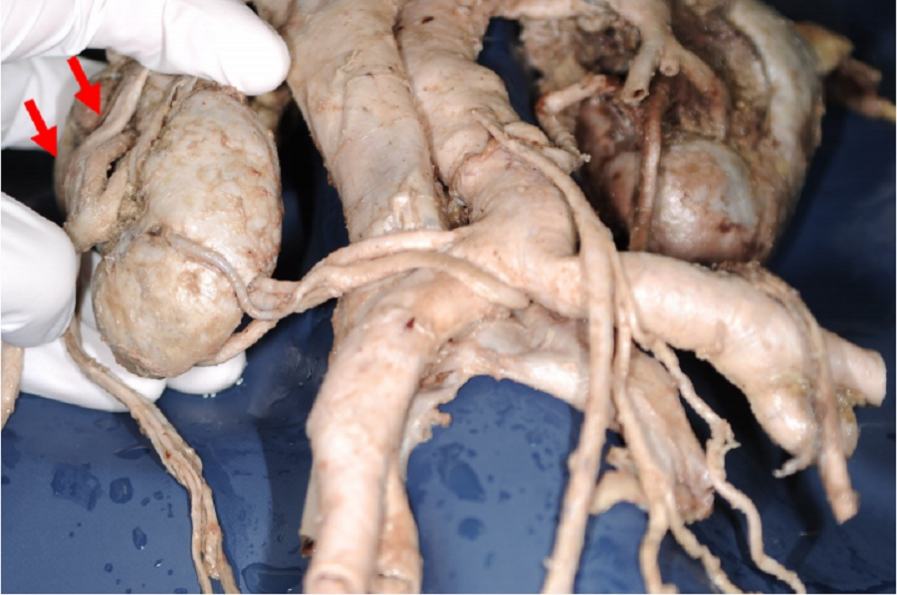
Laterally malrotated kidney. Right renal artery entering the laterally faced hilum (arrows).

However, the kidney is one of the most frequent organs to have variations in shape and position. Variants of the kidney such as a horseshoe kidney (Figure 3), a malrotated kidney (Figure 4) or an ectopic kidney often have aberrant renal arteries [20-22]. Such variant renal vasculature might result in a greater risk of kidney injury during a lateral spine approach. According to Satyapal, et al. [23], approximately 28% of kidneys have accessory renal arteries. Moreover, the course of additional arteries is unpredictable as they can enter the renal hilum either posteriorly or superiorly, or enter directly into the renal parenchyma. Lastly, a retroaortic left renal vein has been detected in approximately 2-4% of the population [24-29] and brings the renal vein closer to the vertebral column and thus closer into the field of an extreme lateral approach to the lumbar spine.

## Conclusions

Our anatomical study analyzed possible kidney injury following an extreme lateral transpsoas approach to the lumbar spine. The results of this study might help surgeons better recognize the potential for kidney injury during such a procedure. A better appreciation of the soft tissues adjacent to the spine can improve patient outcomes following spine surgery. As detailed in this paper, due to the variety of pathologies and anomalies that affect the location of the kidneys relative to the spine, pre-operative imaging should be considered in order to avoid injury during the procedure.
